# Effects of Dexmedetomidine on the Pharmacokinetics of Parecoxib and Its Metabolite Valdecoxib in Beagles by UPLC-MS/MS

**DOI:** 10.1155/2020/1563874

**Published:** 2020-08-06

**Authors:** Jie Hu, Bing-feng Lv, Wen-jing Guo, Bo-wen Wang, Di Miao, Xiang-jun Qiu, Xing-peng Chen

**Affiliations:** ^1^Luoyang Central Hospital Affiliated to Xinxiang Medical University, Luoyang 471003, China; ^2^Luoyang Central Hospital, Henan Province, Luoyang 471003, China; ^3^School of Basic Medicine, Henan University of Science and Technology, Luoyang 471023, China

## Abstract

A sensitive and reliable ultraperformance liquid chromatography tandem mass spectrometry (UPLC-MS/MS) method was developed for the simultaneous determination of parecoxib and its metabolite valdecoxib in beagles. The effects of dexmedetomidine on the pharmacokinetics of parecoxib and valdecoxib in beagles were studied. The plasma was precipitated by acetonitrile, and the two analytes were separated on an Acquity UPLC BEH C18 column (2.1 mm × 50 mm, 1.7 *μ*m); the mobile phase was acetonitrile and 0.1% formic acid with gradient mode, and the flow rate was 0.4 mL/min. In the negative ion mode, the two analytes and internal standard (IS) were monitored by multiple reaction monitoring (MRM), and the mass transition pairs were as follows: *m*/*z* 369.1 → 119.1 for parecoxib, *m*/*z* 313.0 → 118.0 for valdecoxib, and *m*/*z* 380.0 → 316.0 for celecoxib (IS). Six beagles were designed as a double cycle self-control experiment. In the first cycle, after intramuscular injection of parecoxib 1.33 mg/kg, 1.0 mL blood samples were collected at different times (group A). In the second cycle, the same six beagles were intravenously injected with 2 *μ*g/kg dexmedetomidine for 7 days after one week of washing period. On day 7, after intravenous injection of 2 *μ*g/kg dexmedetomidine for 0.5 hours, 6 beagle dogs were intramuscularly injected with 1.33 mg/kg parecoxib, and blood samples were collected at different time points (group A). The concentration of parecoxib and valdecoxib was detected by UPLC-MS/MS, and the main pharmacokinetic parameters were calculated by DAS 2.0 software. Under the experimental conditions, the method has a good linear relationship for both analytes. The interday and intraday precision was less than 8.07%; the accuracy values were from -1.20% to 2.76%. *C*_max_ of parecoxib in group A and group B was 2148.59 ± 406.13 ng/mL and 2100.49 ± 356.94 ng/mL, *t*_1/2_ was 0.85 ± 0.36 h and 0.85 ± 0.36 h, and AUC_(0‐*t*)_ was 2429.96 ± 323.22 ng·h/mL and 2506.38 ± 544.83 ng·h/mL, respectively. *C*_max_ of valdecoxib in group A and group B was 2059.15 ± 281.86 ng/mL and 2837.39 ± 276.78 ng/mL, *t*_1/2_ was 2.44 ± 1.55 h and 2.91 ± 1.27 h, and AUC_(0‐*t*)_ was 4971.61 ± 696.56 ng·h/mL and 6770.65 ± 453.25 ng·h/mL, respectively. There was no significant change in the pharmacokinetics of parecoxib in groups A and B. *C*_max_ and AUC_(0 − ∞)_ of valdecoxib in group A were 37.79% and 36.19% higher than those in group B, respectively, and *t*_1/2_ was increased from 2.44 h to 2.91 h. *V*_*z*_/*F* and CL_*z*_/*F* were correspondingly reduced, respectively. The developed UPLC-MS/MS method for simultaneous determination of parecoxib and valdecoxib in beagle plasma was specific, accurate, rapid, and suitable for the pharmacokinetics and drug-drug interactions of parecoxib and valdecoxib. Dexmedetomidine can inhibit the metabolism of valdecoxib in beagles and increase the exposure of valdecoxib, but it does not affect the pharmacokinetics of parecoxib.

## 1. Introduction

Dexmedetomidine is a highly selective *α*2-adrenoceptor agonist with sedative, antianxiety, sympathetic, and analgesic effects [1]. In clinical treatment, dexmedetomidine is mainly administered through continuous intravenous infusion. Due to the risk of respiratory depression and apnea in some cases, it is necessary to monitor patients without intubation [2]. At present, dexmedetomidine has been licensed in Europe and the United States for analgesia of ventilated patients in the adult intensive care unit (ICU) and programed sedation of nonventilated adults [3]. Evidence suggests that dexmedetomidine can be used as a local anesthetic to enhance local anesthesia and provide sedation [4].

After intravenous administration, dexmedetomidine quickly distributes beyond total body water. Dexmedetomidine is metabolized by glucuronization and CYP2A6 hydroxylation and then excreted almost completely as a metabolite in urine [5, 6]. Body weight, liver damage, plasma albumin, and cardiac output may have significant effects on the pharmacokinetics of dexmedetomidine [3].

**Figure 1:**
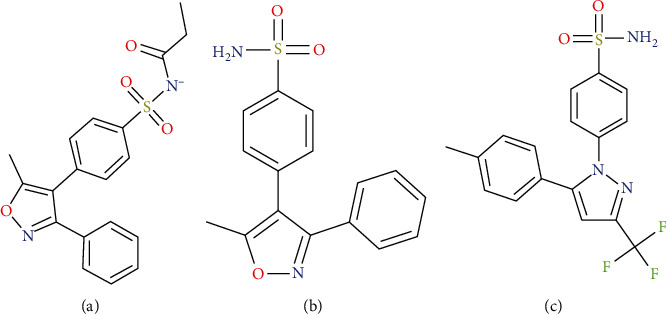
The chemical structure of two analytes and IS: (a) parecoxib; (b) valdecoxib; (c) IS.

Parecoxib (Figure 1(a)) is a water-soluble prodrug of a second-generation cycloxygenase-2- (COX-2-) specific inhibitor and the first of such agent to be developed for injectable use in human medicine. It is a kind of inactive ester amide prodrug, which is rapidly converted to valdecoxib (Figure 1(b)), a specific compound of COX-2, by enzymatic hydrolysis of the liver [7]. The elimination of valdecoxib is widely carried out in the liver in many ways, including cytochrome P450 3A4 (CYP3A4) and CYP2C9 isoenzyme metabolism and sulfaglucosylation (about 20%) [8, 9].

In particular, parecoxib was evaluated in pre- and postoperative treatments of gynaecological, ophthalmological, and colorectal surgery and intracranial and digestive tract tumour resection [10–13].

The perioperative use of parecoxib can significantly improve the effectiveness of postoperative pain after transcatheter arterial chemoembolization (TACE), and parecoxib is effective and well tolerated in relieving pain caused by acute renal colic [14, 15].

Dexmedetomidine and parecoxib are commonly used assistant anesthesia drugs in the clinic, and dexmedetomidine is often used in combination with methoxyphene, midazolam, dezocine, nonsteroidal anti-inflammatory drugs (NSAIDs), and other drugs for analgesia of anesthesia [16, 17]. Dexmedetomidine can enhance its effect when combined with other sedatives and anesthetics. No related pharmacokinetic (PK) interactions were observed with dexmedetomidine (target concentrations were 0.2 to 0.6 ng/mL) in combination with propofol, midazolam, isoflurane, or fentanyl [4]. However, in one case report, it was found that tacrolimus concentration increased fourfold after dexmedetomidine infusion, which was believed to be caused by CYP3A4 inhibition [18]. The use of antidepressants may be related to the changes of PK and/or pharmacodynamics (PD) of dexmedetomidine, thus enhancing the sedative effect [19].

Dexmedetomidine is a strong inhibitor of cytochrome P450 enzyme [20]. Valdecoxib, the active metabolite of parecoxib, is metabolized by CYP3A4 and CYP2C9 [21]. Therefore, drug-drug interaction (DDI) based on CYP450 may occur in the combination of the two drugs. In the current research, at first, a fast and sensitive UPLC-MS/MS method for simultaneous determination of parecoxib and valdecoxib was developed; celecoxib was used as an internal standard (IS, Figure 1(c)); then, the effects of dexmedetomidine on the pharmacokinetics of parecoxib and valdecoxib in beagles were studied.

## 2. Experimental

### 2.1. Chemicals and Reagents

Parecoxib (purity > 98.0%), valdecoxib (purity > 98.0%), and celecoxib (purity > 98.0%, IS) were purchased from Sigma (St. Louis, MO, USA). Methanol and acetonitrile of LC-grade were provided by Tianjin Kermel Chemical Reagent Co., Ltd. Ultrapure water (resistance > 18 m*Ω*) was prepared by a Millipore Milli-Q purification system.

Parecoxib injection (Dynastat, LOT NO.: w19371) was provided by Pharmacia and Upjohn company. Dexmedetomidine hydrochloride injection (LOT NO.: 180222BP) was obtained from Hengrui Medicine Co., Ltd.

### 2.2. Instrumentation and Conditions

The equipment used in this study includes Waters Acquity UPLC instrument (Waters Corp., Milford, MA, USA), XEVO TQD triple quadrupole mass spectrometer (Waters Corp., Milford, MA, USA), ultrapure water equipment (Millipore, Bedford, USA), and electronic analytical balance and vortex instrument.

Parecoxib, valdecoxib, and IS were separated on an Acquity BEH C18 column (2.1 mm × 50 mm, 1.7 *μ*m) by gradient elution with the mobile phase of 0.1% formic acid (A) and acetonitrile (B) at the flow rate of 0.4 mL/min. The gradient program was as follows: 0.00-0.50 min, 90% A; 0.50-1.00 min, 90-10% A; 1.00-2.00 min, 10% A; 2.00-2.10 min, 10-90% A; and 2.10-3.00 min, 90% A. The column temperature was set at 45°C, and the autosampler was conditioned at 4°C. An injection volume of 2 *μ*L was applied for analysis.

The XEVO TQ-S triple quadrupole mass spectrometer was used for mass spectrometric (MS) measurement with an electrospray ionization (ESI) interface in a negative ionization mode. Under the multiple reaction monitoring (MRM) conditions, quantification was achieved with transitions of *m*/*z* 369.1 → 119.1 for parecoxib, *m*/*z* 313.0 → 118.0 for valdecoxib, and *m*/*z* 380.0 → 316.0 for IS, respectively. All data were acquired in the centroid mode by Masslynx V4.1 software (Waters, Milford, MA, USA).

### 2.3. Solutions Ready

Parecoxib, valdecoxib, and celecoxib were prepared at a respective concentration of 1.0 mg/mL by dissolving in methanol as the stock solutions. Various working solutions for the calibration curve and quality control (QC) were obtained in methanol by the gradient dilution of the stock solutions. The final concentrations of the calibration curves were covered by several points as follows: 5, 10, 50, 100, 500, 1000, 2000, and 4000 ng/mL for parecoxib and valdecoxib. As for QC samples, they were made in the same way at three concentration levels (low, medium, and high concentration), and the concentrations of QC samples in plasma were 10, 500, and 3000 ng/mL for parecoxib and valdecoxib. The internal standard (IS) working solution (1000 ng/mL) was also prepared by diluting the stock solution of celecoxib with acetonitrile. All solutions were stored in a refrigerator at -20°C.

### 2.4. Sample Preparation

Before analysis, frozen beagle plasma samples were thawed to room temperature. In a 1.5 mL centrifuge tube, an aliquot of 200 *μ*L of the IS working solution (1000 ng/mL in acetonitrile) was added to 100 *μ*L of a plasma sample. The tubes were vortex mixed for 1.0 min. After centrifugation at 15,000 g for 15 min, the supernatant (2 *μ*L) was injected into the UPLC-MS/MS system for analysis.

### 2.5. Method Validation

UPLC-MS/MS is a common method for the detection of biological samples, which has the advantages of rapidity, sensitivity, and accurateness. The UPLC-MS/MS method has been reported for the simultaneous determination of parecoxib and its metabolite valdecoxib [22, 23]. In this experiment, based on these methods, a rapid, sensitive, and accurate detection method was established and validated for specificity, linearity, precision, accuracy, recovery, and stability. The UPLC-MS/MS method was validated according to the United States Food and Drug Administration (FDA) guidelines [24].

The specificity was assessed by comparing chromatograms of blank beagle plasma samples; blank plasma spiked with parecoxib, valdecoxib, and IS; and a beagle plasma sample 3.0 h after injection of parecoxib.

The calibration curves were constructed and validated by analyzing spiked calibration samples for three days in a row. The linearity of the assay was assessed by analyzing the calibration curves using least-squares linear regression of the peak area ratios of the analytes to the IS versus the nominal concentration of the calibration standard with a weighed factor (1/*χ*^2^). The lower limit of quantification (LLOQ) was selected as the lowest concentration used in the calibration curve. The carryover test was performed by injecting a blank plasma sample injected with parecoxib and valdecoxib (4000 ng/mL) followed by a blank sample.

The accuracy and precision were assessed by the determination of QC samples at three concentration levels (10, 500, and 3000 ng/mL) in six replicates. On the same day, the intraday precision was calculated, and the interday precision was calculated by continuous measurement within 3 days. Precision was defined as the relative standard deviation (RSD, %) and accuracy as the relative error (RE, %).

Parecoxib and valdecoxib extraction recovery was compared by comparing the peak area of the conventional pretreated QC sample pretreatment with the peak area after extracting the corresponding blank plasma concentration (after extraction), and the matrix effect (ME) was evaluated by comparing the peak area of the analyte to water extraction after the sample and the corresponding replacement sample.

Stability studies in biosamples were also conducted at three QC levels in several different storage conditions: room temperature for 12 h, -20°C for at least 4 weeks, after three freeze-thaw cycles (-20 to 25°C), and processed samples at 4°C in an autosampler tray for 12 h. The stock solution stability of parecoxib, valdecoxib, and IS was observed through six repeated tests. The stock solution portion stored at room temperature for 24 hours was compared with the remaining stock solution stored in a -20°C refrigerator to evaluate room temperature stability. The freezing stability was evaluated by comparing the newly prepared stock solution with the stock solution stored in a refrigerator at -20°C for three months.

### 2.6. Animals

Six beagles (half male, half female, weighing 6 ± 2 kg) were obtained from Hubei Yizhicheng Biological Technology Co., Ltd, and the animal certificate was SCXK(hubei)2016-0020. The institutional approval number for the preclinical study of this experiment was 2019100013. The beagles were adapted to the new environment for 7 days in laboratory conditions. Necessary approval from the Institutional Animal Ethics Committee was obtained to carry out the experiments.

### 2.7. Study Design

Before the test, the beagles had free access to water but were fasted for 12 h. Six beagles were designed as a double cycle self-control experiment. Blood samples (1.0 mL) were collected from the forelimb cephalic vein or the small saphenous vein of the hindlimb and collected into heparinized tubes at 0.17, 0.33, 0.67, 1, 1.5, 2, 3, 4, 6, 9, 12, and 24 h after intramuscular injection of parecoxib 1.33 mg/kg in the first cycle (group A). Blood samples were centrifuged for 10 min at 3000 × g, and the plasma was collected and kept frozen at -20°C until analysis, and the samples belonged to group A.

After the one-week washout period, the same six beagles were injected intravenously slowly with 2 *μ*g/kg dexmedetomidine every morning in the second cycle (group B), with continuous injection for 7 days. On the seventh day, the six beagles were given 1.33 mg/kg parecoxib intravenously half an hour after intravenous injection of 2 *μ*g/kg dexmedetomidine. The blood samples (1.0 mL) were collected into heparinized tubes at 0, 0.17, 0.33, 0.67, 1, 1.5, 2, 3, 4, 6, 9, 12, and 24 h after intramuscular injection of parecoxib. Plasma was also separated and preserved, and the samples belonged to group B.

### 2.8. Plasma Sample Detection

The above-developed UPLC-MS/MS method was used to simultaneously detect parecoxib and its active metabolite valdecoxib in the beagle plasma of group A and group B.

### 2.9. Data Analysis

The pharmacokinetic parameters of parecoxib and valdecoxib were calculated by the noncompartmental analysis using the DAS 2.0 software. All data were expressed as the mean ± standard deviation (SD).

## 3. Results

### 3.1. Specificity

**Figure 2:**
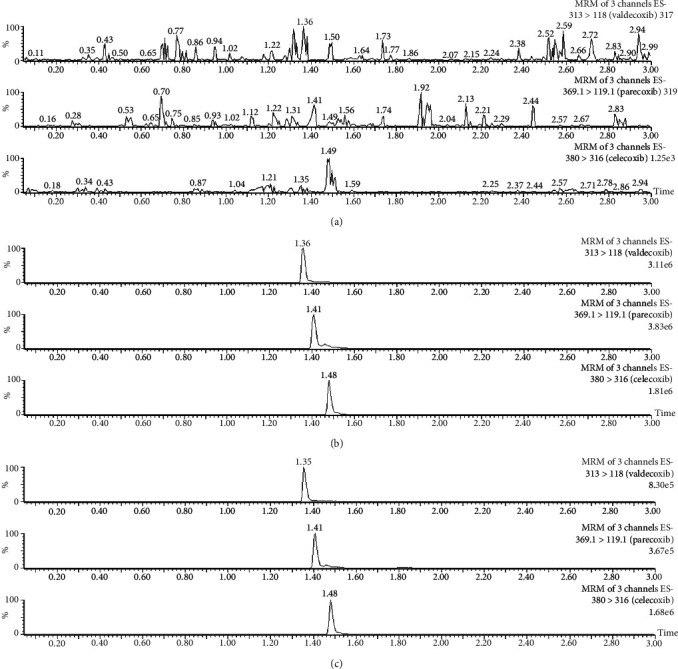
Representative chromatograms in negative ion mode: (a) a blank plasma sample; (b) a blank plasma sample spiked with parecoxib, valdecoxib, and IS; (c) a beagle plasma sample 3.0 h after injection of parecoxib.

Under the above experiment, parecoxib, valdecoxib, and IS were well separated from endogenous substances. Representative chromatograms of a blank plasma sample (a), a blank plasma sample spiked with two analytes and IS (b), and a beagle sample (c) are shown in Figure 2. Dexmedetomidine and plasma endogenous substances did not interfere with the detection of the two analytes. The mean retention times of parecoxib, valdecoxib, and celecoxib (IS) were 1.41, 1.36, and 1.48 min, respectively. The total running time for each sample was 3.0 min.

### 3.2. Linearity and Carryover

In the concentration range of 5–4000 ng/mL, the typical regression equations of parecoxib and valdecoxib were *y* = 1.5 × 10^−3^ *x* − 1.34 × 10^−2^ (*r* = 0.9993) and *y* = 1.8 × 10^−3^ *x*–3.82 × 10^−2^ (*r* = 0.9991), respectively, where *y* represents the peak area ratio and *x* represents the plasma concentration. The lower limit of quantification (LLOQ) of parecoxib and valdecoxib was 5 ng/mL. Carryover test results show that in the UPLC-MS/MS analysis, carryover does not affect the determination of parecoxib and valdecoxib.

### 3.3. Precision and Accuracy

Table 1 shows the results obtained for the intraday and interday precision and accuracy of parecoxib and valdecoxib. The precision (% RSD) for the two analytes under investigation did not exceed 8.07%. Accuracy (% RE) for all analytes was in the range from -1.20% to 2.76% at the concentrations studied and met the requirements of validation.

### 3.4. Recovery and ME

The recovery and ME results were investigated and are shown in Table 2, which were accepted in accordance with the US FDA guidelines for the method validation to differentiate and qualify the analytes in a sample. The recoveries of the two analytes were higher than 80%. No ME was observed to influence the determination of two analytes in beagle plasma.

### 3.5. Stability and Stock Solution Stability

All results for the stability samples tested are summarized in Tables 3 and 4, and they were within the acceptable criteria of ±15%, indicating that parecoxib and valdecoxib were stable under the four conditions described.

### 3.6. Effects of Dexmedetomidine on Pharmacokinetics of Parecoxib and Valdecoxib

**Figure 3:**
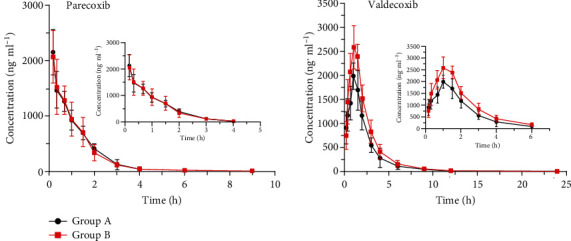
The mean plasma concentration-time curve profile of parecoxib and valdecoxib in group A and group B.

The plasma concentration-time curve of parecoxib of group A and group B in six beagles after a single intramuscular injection of parecoxib is shown in Figure 3. The plasma concentration-time curve of valdecoxib of group A and group B in six beagles after 7 days of intravenous administration of dexmedetomidine and then administration of parecoxib is shown in Figure 3 too.

The DAS 2.0 program was used to calculate the main pharmacokinetic parameters of parecoxib and valdecoxib, including *t*_1/2_, *C*_max_, *T*_max_, *V*_*z*_/*F*, CL_*z*_/*F*, AUC_(0 − *t*)_, and AUC_(0 − ∞)_. Meanwhile, Table 5 shows the main pharmacokinetic parameters of parecoxib and valdecoxib.

Results show that after intramuscular injection of parecoxib, parecoxib was absorbed rapidly, and then, it was transformed into valdecoxib, an active metabolite. There was no significant change in the pharmacokinetics of parecoxib in groups A and B, it was suggested that dexmedetomidine does not affect the conversion of parecoxib. *C*_max_ and AUC_(0 − ∞)_ of valdecoxib in group A were 37.79% and 36.19% higher than those in group B, respectively, and *t*_1/2_ was increased from 2.44 h to 2.91 h. *V*_*z*_/*F* and CL_*z*_/*F* were correspondingly reduced, respectively. It was suggested that dexmedetomidine can inhibit the metabolism of valdecoxib and increase the exposure of valdecoxib in beagles.

## 4. Discussion

This study established a new UPLC-MS/MS method that can simultaneously detect the content of beagle dog plasma parecoxib and its metabolite valdecoxib. This method fully complies with bioassay standards, with high sensitivity and short analysis time (3 min). In the choice of plasma processing methods, we directly precipitate proteins with acetonitrile. The operation method is simple and fast, and the detection results are reliable. At the same time, the endogenous substances in the beagle dog plasma sample do not affect the determination of the analyte.

When the pharmacokinetics (PKs) and pharmacodynamics (PDs) of a drug are changed by one or more drugs, drug-drug interactions (DDI) will occur [25]. It is important to assess the risks of DDI, because DDI can significantly affect patient safety [26]. Pharmacokinetics (PKs) can occur at any stage of the body process, such as absorption, distribution, metabolism, and excretion, but primarily at the metabolic stage of the drug [27]. The hepatic cytochrome P450 (CYP) enzyme system is the main site where drug metabolism and 86% drug interaction occur.

Nonsteroidal anti-inflammatory drugs, or NSAIDs, play antipyretic, analgesic, and anti-inflammatory effects by inhibiting COX and reducing the synthesis of prostaglandins. NSAIDs are available as oral tablets and solutions [28]. Metabolism of NSAIDs is controlled by the CYP2C family of enzymes. Therefore, the genetic variation of these enzymes has a great influence on the therapeutic effect of NSAIDs. CYP2C9 is a major factor in the enzymatic degradation and drug clearance of some NSAIDs [29]. Studies have shown that the CYP2C9∗3 mutation in particular results in slower metabolism of these compounds; thus, it has a stronger therapeutic effect on individuals with this polymorphism [30].

Parecoxib was rapidly and almost completely converted to valdecoxib. The elimination of valdecoxib was extensively carried out in a variety of ways in the liver, including cytochrome CYP3A4 and CYP2C9 enzyme metabolism and sulfonamide glucose hydroformylation (about 20%) [21].

Dexmedetomidine is a highly selective *α*2-adrenoceptor agonist with sedative, antianxiety, sympathetic, and analgesic effects [1]. Dexmedetomidine is metabolized by glucuronization and CYP2A6 hydroxylation and then excreted almost completely as a metabolite in urine [5, 6]. Dexmedetomidine is a strong inhibitor of cytochrome CYP450 enzyme [20]. The combination of sufentanil and dexmedetomidine can cause drug-drug interactions, which may promote the sedation and prolong the respiratory depression by increasing the exposure level of dexmedetomidine brain tissue. In clinical application, attention should be paid to the possible drug-drug interactions or adverse reactions caused by the combination of these two drugs [20]. Dexmedetomidine enhances the effect of mepivacaine on reducing local blood flow, prolonging the tissue retention time, and increasing the local anesthetic effect without affecting the hemodynamics of local administration [31].

The results of this study show that there was no significant change in the pharmacokinetics of parecoxib in groups A and B; it was suggested that dexmedetomidine does not affect the conversion of parecoxib in beagles. *C*_max_ and AUC_(0 − ∞)_ of valdecoxib in group A were 37.79% and 36.19% higher than those in group B, respectively, and *t*_1/2_ was increased from 2.44 h to 2.91 h. *V*_*z*_/*F* and CL_*z*_/*F* were correspondingly reduced, respectively. It was suggested that dexmedetomidine can inhibit the metabolism of valdecoxib and increase the exposure of valdecoxib in beagles.

Although there are some differences in the pharmacokinetics of different species, the results of animal experiments can provide some reference for clinical medication [32]. Therefore, clinically, when dexmedetomidine is combined with parecoxib, we should not only pay attention to the enhancement of the analgesic effect caused by the combination but also pay attention to the adverse drug reactions caused by drug-drug interactions.

## 5. Conclusions

The developed UPLC-MS/MS method for simultaneous determination of parecoxib and its metabolite valdecoxib in beagle plasma was specific, accurate, rapid, and suitable for the pharmacokinetics and drug-drug interactions of parecoxib and valdecoxib. Dexmedetomidine can inhibit the metabolism of valdecoxib in beagles and increase the exposure of valdecoxib, but it does not affect the pharmacokinetics of parecoxib.

## Figures and Tables

**Table 1:** Precision and accuracy of two analytes in beagle plasma (*n* = 6, mean ± SD).

Compound	Added (ng/mL)	Intraday			Interday		
		Found (ng/mL)	RSD (%)	RE (%)	Found (ng/mL)	RSD (%)	RE (%)
Parecoxib	10	9.88 ± 0.72	7.27	-1.20	9.97 ± 0.81	8.07	-0.30
	500	513.80 ± 28.16	5.48	2.76	503.43 ± 34.20	6.79	0.69
	3000	3061.46 ± 103.32	3.34	2.05	3028.77 ± 107.85	3.56	0.96
Valdecoxib	10	9.94 ± 0.64	6.47	-0.60	10.07 ± 0.72	7.17	0.70
	500	497.05 ± 24.75	4.98	-0.59	491.68 ± 31.90	6.49	1.66
	3000	3079.67 ± 122.17	3.97	2.66	3045.14 ± 105.25	3.46	1.50

**Table 2:** The recoveries and ME of two analytes in beagle plasma (*n* = 6, mean ± SD).

Compound	Added (ng/mL)	Recovery (%)	RSD (%)	ME (%)	RSD (%)
Parecoxib	10	81.69 ± 2.83	3.47	100.60 ± 7.79	7.74
	500	84.41 ± 2.33	2.76	101.55 ± 6.18	6.09
	3000	86.47 ± 2.11	2.44	99.52 ± 2.69	2.70
Valdecoxib	10	82.19 ± 2.01	2.44	98.63 ± 5.47	5.55
	500	84.16 ± 3.01	3.58	98.95 ± 4.08	4.13
	3000	85.72 ± 2.76	3.22	102.04 ± 3.72	3.64

**Table 3:** The stability of two analytes in beagle plasma (*n* = 6, mean ± SD).

Compounds	Added (ng/mL)	Room temperature, 12 h		Autosampler 4°C, 12 h		Three freeze-thaw		-20°C, 4 weeks	
		RSD (%)	RE (%)	RSD (%)	RE (%)	RSD (%)	RE (%)	RSD (%)	RE (%)
Parecoxib	10	5.32	-2.55	4.35	0.67	6.97	3.58	4.68	3.43
	500	3.08	-1.20	4.79	-0.80	4.83	-1.31	4.62	2.72
	3000	1.60	1.23	1.72	1.75	3.54	0.55	2.53	-2.08
Valdecoxib	10	4.34	2.63	4.59	1.38	4.37	3.18	2.39	-5.15
	500	5.17	1.56	5.71	-1.31	6.87	3.03	5.29	1.13
	3000	2.19	-1.19	2.25	0.98	2.14	-1.36	1.44	2.49

**Table 4:** The stock solution stability of parecoxib, valdecoxib, and IS in beagle plasma (*n* = 6).

Compounds	Spiked (*μ*g/mL)	Room temperature, 12 h		-20°C, 3 weeks	
		RSD (%)	RE (%)	RSD (%)	RE (%)
Parecoxib	10	3.80	-0.67	4.13	0.83
Valdecoxib	10	3.43	0.33	4.31	-2.17
IS	10	3.95	-2.17	4.20	-1.83

**Table 5:** Pharmacokinetic parameters of parecoxib and valdecoxib in group A and group B (*n* = 6, mean ± SD).

Parameters	Parecoxib		Valdecoxib	
	Group A	Group B	Group A	Group B
*t*_1/2_ (h)	0.85 ± 0.36	0.88 ± 0.33	2.44 ± 1.55	2.91 ± 1.27
*T*_max_ (h)	0.17 ± 0.00	0.20 ± 0.07	1.17 ± 0.26	1.17 ± 0.26
*C*_max_ (ng/mL)	2148.59 ± 406.13	2100.49 ± 356.94	2059.15 ± 281.83	2837.39 ± 276.78
*V*_*z*_/*F* (L/kg)	0.56 ± 0.08	0.55 ± 0.12	0.27 ± 0.04	0.20 ± 0.01
CL_*z*_/*F* (L/h/kg)	0.66 ± 0.22	0.67 ± 0.23	0.98 ± 0.70	0.83 ± 0.35
AUC_(0 − *t*)_ (ng·h/mL)	2429.96 ± 323.22	2506.38 ± 544.83	4971.61 ± 696.56	6770.65 ± 453.25
AUC_(0 − ∞)_ (ng·h/mL)	2433.98 ± 326.42	2510.94 ± 545.53	4989.92 ± 691.58	6787.47 ± 454.04

## Data Availability

The data used to support the findings of this study are available from the corresponding author upon request.
